# 4531 Setting the Stage for Research Success: Creation of Standardized Physician-Scientist Training Program Guidelines to Facilitate Research During Clinical Training

**DOI:** 10.1017/cts.2020.222

**Published:** 2020-07-29

**Authors:** Stephanie A. Freel, Katherine Barrett, Jillian Hurst, Rasheed Gbadegesin, Sallie Permar

**Affiliations:** 1 Duke University

## Abstract

OBJECTIVES/GOALS: To ameliorate the leaky pipeline of physician-scientists, we must address the factors that cause medical trainees to disengage from research. Here we describe the development of standardized Physician-Scientist Training Program guidelines that may be implemented across disciplines to address these challenges. METHODS/STUDY POPULATION: Maintenance of a robust pool of physician-scientists is critical to meet the rapidly growing need for novel therapeutics. A variety of factors contribute to the decline of this pool. Key among these are a lengthy training period that segregates research from clinical training, thus impeding research progress and milestones that allow for a successful research career. Through engagement of residency program directors and Vice Chairs of Research, we have created a series of guidelines that promote residency research tracks and enable better integration of research and clinical training time. Guidelines have been piloted in the Departments of Pediatrics, Medicine and Surgery in the context of 2 new R38-supported programs. RESULTS/ANTICIPATED RESULTS: Our physician-Scientist Training Program (PSTP) guidelines were developed by our central Office of Physician-Scientist Development (OPSD) after a successful pilot of an integrated research residency program in the Department of Pediatrics [Duke Pediatric Research Scholars (DPRS); Hurst, et al, 2019], which has included 36 resident and fellow scholars over 3 years. To date, eight clinical departments have adopted our PSTP guidelines as part of their R38-supported or pending programs. The OPSD has recently created a tracking database for scholar metrics, which will further promote PSTP development by enabling centralized reporting on scholar success to individual programs. DISCUSSION/SIGNIFICANCE OF IMPACT: PSTP guidelines enable effective implementation of new programs by sharing best practices and lessons learned, standardizing expectations, and defining metrics of success. By promoting proven strategies for integrated clinical and research training, PSTP guidelines may aid in retaining trainees pursuing research careers.

